# Novel use of cholangioscopy-assisted enteroscopy for foreign body removal from a narrow-ostium ileal diverticulum

**DOI:** 10.1055/a-2767-0598

**Published:** 2026-01-22

**Authors:** Jing Guo, Yanbo Yu, Rui Ji, Jun Liu, Xiu-Li Zuo

**Affiliations:** 1 Department of Gastroenterology, Qilu Hospital, Shandong University, Jinan, China

**Fig. 1:**
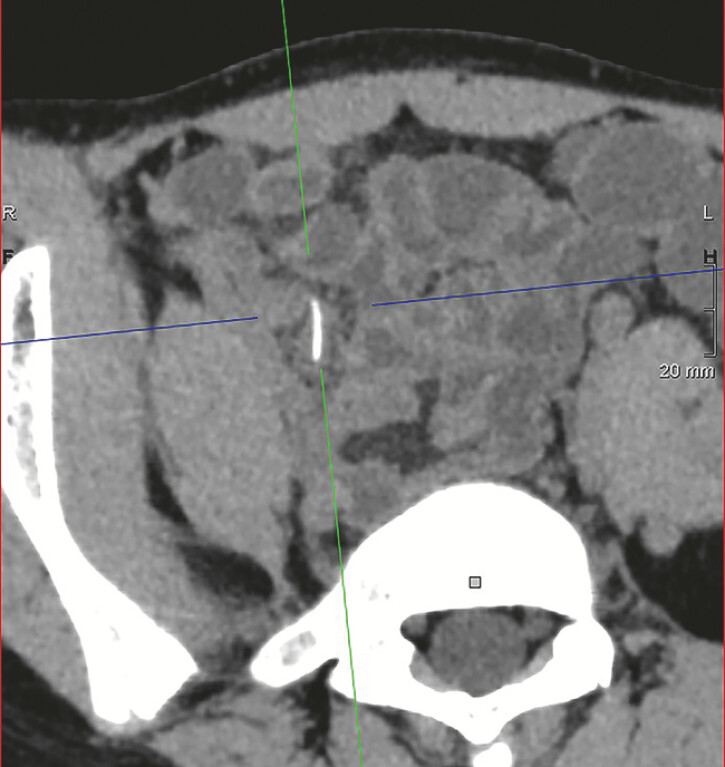
A computed tomographic image showing a high-density shadow in the right lower quadrant of abdomen, indicating an intestinal foreign body.

**Fig. 2:**
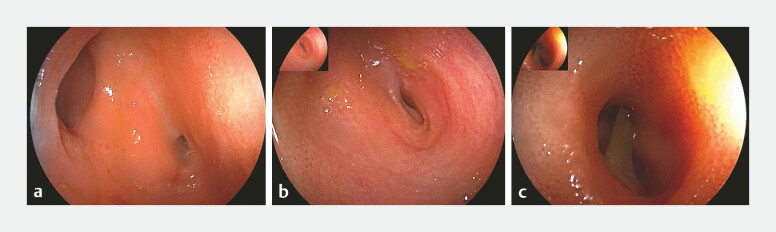
An image from double-balloon enteroscopy: **a** the “double lumen” sign at the ileum about 100 cm beyond the ileocecal valve; **b** the narrow opening of the ileal diverticulum; **c** a bone foreign body in the diverticulum, and the inner wall of diverticulum cannot be observed.

**Fig. 3:**
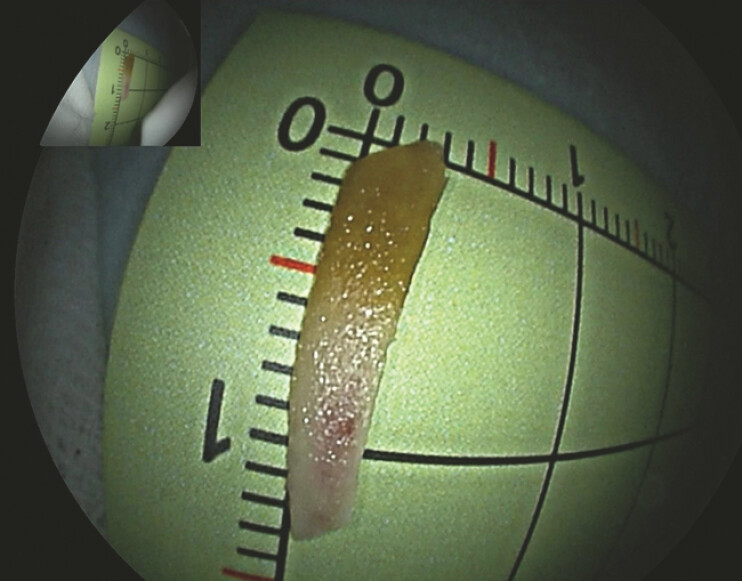
Photograph of the extracted foreign object, which was found to be a 1.4-cm chicken bone.

**Fig. 4:**
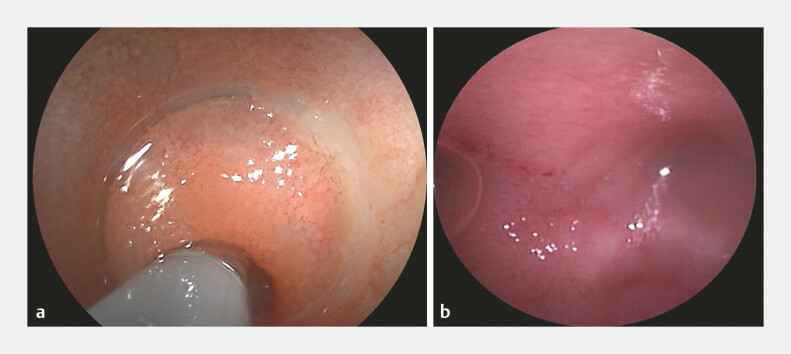
Image of the digital single-operator cholangioscope: **a** Intubation of the diverticulum with the cholangioscope. **b** Observing the condition of the inner wall of the diverticulum.

A 13-year-old girl was presented with a 20-day history of right-sided abdominal pain. She denied consuming any special foods. Computed tomography (CT) revealed a high-density focus in the right lower abdomen, suggestive of an intestinal foreign body ( Fig. 1 ). Given the high surgical risk and uncertain prognosis, double-balloon enteroscopy was performed via a retrograde approach. At the site 100 cm proximal to an ileocecal valve, a “double lumen” sign was observed ( Fig. 2 **a** ), comprising the intestinal lumen and a narrow-opening diverticulum ( Fig. 2 **b** ). Endoscopic visualization identified a rod-like foreign body embedded within the diverticulum ( Fig. 2 **c** ). A conical transparent cap was attached to the enteroscopy tip to facilitate retrieval. With the cap stabilized at the orifice, the foreign body was successfully retrieved using biopsy forceps, which confirmed a 1.4 cm chicken bone ( Fig. 3 ). Post-retrieval bleeding was noted; however, the narrow opening limited further visualization. For detailed assessment, a digital single-operator cholangioscope was inserted into the diverticulum cavity ( Fig. 4 **a** ), providing clear visualization of a short cavity with mucosal edema and minor blood clots. After irrigation, no residual foreign body, active bleeding, or perforation was confirmed. ( Fig. 4 **b** , Video 1 ).

Removal of a foreign body from an ileal narrow-opening diverticulum via enteroscopy assisted with a digital single-operator cholangioscope.Video 1

The extraction of sharp foreign bodies from the small intestine is technically challenging with enteroscopic techniques 1 . This difficulty is further exacerbated when the foreign body is lodged within a diverticulum featuring a narrow ostium, which precludes direct visualization. The cholangioscopy 2 can be navigated through these confined openings to effectively address this scenario. To the best of our knowledge, this is the first reported case in which a foreign body was successfully retrieved from an ileal diverticulum with a narrow ostium. Additionally, it marks the novel application of cholangioscopy in conjunction with double-balloon enteroscopy. This integrated approach provided a minimally invasive alternative, avoiding the need for surgery.

Endoscopy_UCTN_Code_TTT_1AP_2AD
